# Temporal Changes in Diagnostic Composition and Treatment Activity Among Mechanically Ventilated ICU Patients Receiving Hyperbaric Oxygen Therapy: An 11-Year Single-Centre Retrospective Study

**DOI:** 10.3390/jcm15187089

**Published:** 2026-09-12

**Authors:** Aneta Miszewska, Olga Sobczak, Piotr Góralczyk, Jacek Kot

**Affiliations:** 1 Division of Anaesthesiology Nursing & Intensive Care, Medical University of Gdańsk, ul. Dębinki 7, Building 15, 80-211 Gdansk, Poland; 2 Department of Hyperbaric Medicine and Sea Rescue, University Centre for Maritime and Tropical Medicine, ul. Powstania Styczniowego 9b, 81-519 Gdynia, Polandjkot@gumed.edu.pl (J.K.); 3 National Centre for Hyperbaric Medicine, Institute of Maritime and Tropical Medicine, Medical University of Gdańsk, ul. Powstania Styczniowego 9b, 81-519 Gdynia, Poland

**Keywords:** hyperbaric oxygen therapy, intensive care unit, mechanical ventilation, necrotising soft-tissue infection, carbon monoxide poisoning, temporal trends

## Abstract

**Background**: Mechanically ventilated intensive care patients receiving hyperbaric oxygen therapy (HBOT) are uncommon and resource-intensive. We assessed temporal changes over 11 years in diagnostic composition, HBOT treatment activity, organ-support requirements, and in-hospital mortality at an academic centre. **Methods**: This single-centre retrospective cohort included mechanically ventilated ICU patients receiving HBOT during the period 2013–2023. Diagnoses were grouped as carbon monoxide (CO) poisoning, clostridial myonecrosis/gas gangrene, non-clostridial necrotising soft-tissue infections (NSTIs), or other indications. Annual patient and HBOT patient-session volumes and the annual number and proportion of nocturnal sessions were summarised descriptively. Calendar-time associations were estimated using exploratory univariable logistic regression with calendar year entered as a continuous predictor and are reported as odds ratios (ORs) per one-year increase with 95% confidence intervals (CIs). **Results**: Of 6410 HBOT-treated patients, 176 mechanically ventilated ICU patients (2.7%) underwent 1381 sessions; 781 (56.6%) were nocturnal. Annual volumes ranged from 10 to 22 patients and from 66 to 190 sessions, without a monotonic increase. The estimated OR per one-year increase was 0.85 (95% CI: 0.76–0.95) for CO poisoning, 0.85 (95% CI: 0.76–0.96) for clostridial myonecrosis/gas gangrene, 1.23 (95% CI: 1.11–1.36) for non-clostridial NSTIs, and 1.17 (95% CI: 1.04–1.31) for catecholamine infusion. **Conclusions**: Diagnostic composition shifted towards non-clostridial NSTIs. Catecholamine infusion was more frequent in the later calendar years, whereas the CVVHDF estimate was small and imprecise and the mortality estimate was near null. Persistent nocturnal activity supports ICU-capable infrastructure and flexible staffing for emergency HBOT.

## 1. Introduction

Mechanically ventilated intensive care unit (ICU) patients undergoing hyperbaric oxygen therapy (HBOT) constitute a small but clinically demanding subgroup within hyperbaric medicine [1]. HBOT is recognised for selected acute indications that may occur in critically ill patients; these include carbon monoxide poisoning, air or arterial gas embolism, severe decompression sickness, clostridial gas gangrene, non-clostridial necrotising soft-tissue infections, and acute crush injury or other acute traumatic ischaemias [2]. In infectious indications, HBOT remains adjunctive to surgical source control and antimicrobial treatment rather than a replacement for standard care [3]. In ICU patients, the decision to use HBOT should be based on an individual risk–benefit assessment and should not delay or interrupt overall critical-care management [2]. Only a minority of hyperbaric centres have ICU-level staffing, specialised equipment, a 24/7 schedule, and experience in treating critically ill patients [4]. Therefore, delivery of HBOT to mechanically ventilated ICU patients requires infrastructure and expertise beyond routine hyperbaric practice [2,4].

The operational complexity of ICU-HBOT is substantial. Continuation of ICU-level monitoring and treatment during HBOT requires a chamber designed and equipped for critically ill patients, ideally located in or near the ICU [2]. Chamber-compatible ventilators, monitoring systems, infusion pumps, suction/drainage systems, and other life-support devices are central to safe hyperbaric critical care, whereas complex technologies such as haemofiltration, cardiac assist devices, and extracorporeal membrane oxygenation may be incompatible with the hyperbaric environment [5]. Mechanically ventilated patients may require controlled ventilation, sedation, and mitigation of patient–ventilator asynchrony or barotrauma risk during HBOT [2,6]. Staff caring for critically ill patients during HBOT should be trained in both emergency or intensive care and hyperbaric medicine, and critically ill patients commonly require one-to-one care inside the chamber [7]. Recent single-centre ICU-HBOT data identified catecholamine and analgosedation infusions, nocturnal sessions, pleural drainage, and mechanical ventilation via an endotracheal tube as factors relevant to transport and HBOT-session risk [1]. These requirements have direct implications for staffing models, chamber access, the provision of nocturnal HBOT, patient-safety procedures, and coordination between intensive care, surgical, emergency, and hyperbaric teams [1,2,5,7].

Existing publications on ICU-HBOT have mainly addressed safety in mechanically ventilated patients [1,2,4], transport feasibility [1,8], equipment constraints [5], staffing requirements [7], or indication-specific recommendations [3]. Mechanically ventilated and critically ill patients can undergo HBOT in experienced centres, but this evidence is largely operational rather than epidemiological [1,6,8]. Large disease-specific datasets exist for necrotising soft-tissue infections and carbon monoxide poisoning, including nationwide or multicentre cohorts [9,10,11]. However, these disease-specific studies do not describe long-term temporal changes in the distribution of diagnostic categories or HBOT treatment activity in cohorts of mechanically ventilated ICU-HBOT patients treated for multiple indications. Carbon monoxide poisoning and necrotising soft-tissue infections were considered within the same service-defined cohort because both are recognised acute indications for HBOT and severe presentations may require continuation of ICU-level care during treatment [2,3,4]. For carbon monoxide poisoning specifically, European consensus recommends HBOT for patients with altered consciousness or clinical neurological, cardiac, respiratory, or psychological manifestations, irrespective of the carboxyhaemoglobin level at hospital admission, as well as for pregnant patients irrespective of clinical presentation or carboxyhaemoglobin level (Type 1 recommendation, Level B evidence) [3]. Eligibility for HBOT is distinct from the need for ICU-level care; severe carbon monoxide poisoning may cause neurological impairment, respiratory failure, or haemodynamic instability requiring continuous monitoring and organ support, including invasive mechanical ventilation and, when indicated, vasoactive therapy [2,3,4]. Because the centre provides emergency and inpatient HBOT for patients with different recognised indications, cohort membership was determined independently of diagnosis by hospitalisation, receipt of at least one HBOT session, and a requirement for invasive mechanical ventilation; diagnoses were retained as mutually exclusive analytical categories because their treatment schedules and resource requirements differ.

Accordingly, general HBOT registries and disease-specific cohorts describe important aspects of hyperbaric practice, but they do not quantify the volume and timing of HBOT delivered to the small subgroup of hospitalised patients who require invasive mechanical ventilation and ICU-compatible HBOT. This distinction is important because patterns of HBOT provision may change even when annual patient counts remain variable or relatively stable: indication, repeated sessions, nocturnal treatment, vasoactive support, transport, and multidisciplinary coordination impose different clinical and operational requirements. In this study, diagnostic composition refers to the distribution of patients across four mutually exclusive categories: carbon monoxide poisoning; clostridial myonecrosis/gas gangrene; non-clostridial necrotising soft-tissue infections (other NSTIs, including Fournier’s gangrene); and other HBOT indications. The present study therefore evaluated temporal changes over eleven years in diagnostic composition and treatment activity among mechanically ventilated ICU patients receiving HBOT at a tertiary academic hyperbaric centre. We specifically assessed annual patient and HBOT patient-session volumes, the annual number and proportion of nocturnal patient-sessions, organ-support variables, and in-hospital mortality between 2013 and 2023.

## 2. Materials and Methods

### 2.1. Study Design

This was a retrospective, single-centre observational study with a secondary temporal analysis of mechanically ventilated intensive care patients treated with HBOT. The study covered eleven complete consecutive calendar years, from 1 January 2013 through 31 December 2023. The study was designed as an epidemiological and service-activity analysis. It was not designed to evaluate the therapeutic efficacy of HBOT or to establish causal relationships between calendar period, referral patterns, health-system changes, or patient outcomes.

### 2.2. Study Setting

The study was conducted at the Department of Hyperbaric Medicine and Sea Rescue, University Centre for Maritime and Tropical Medicine in Gdynia, Poland (HC)—an academic hyperbaric centre providing both inpatient and outpatient HBOT. Its organisational model, integrating intensive care and hyperbaric treatment pathways, was described previously [1]. Care is delivered in accordance with recognised European standards for hyperbaric medicine and critical-care HBOT practice [2].

Critically ill patients requiring repeated HBOT are admitted to the centre rather than transported from other hospitals for each session. The inpatient intensive-care area and hyperbaric chamber are located within the same building, enabling intra-departmental transfer. Patient preparation, transport, chamber care, and post-session management are provided by a single clinical team trained in intensive care and hyperbaric medicine. A medical team and hyperbaric technician are available on site 24/7, allowing both emergency HBOT and scheduled treatment of hospitalised patients.

Because routine outpatient HBOT is also performed during daytime hours, some intensive care–HBOT (ICU-HBOT) sessions in hospitalised patients take place during the night shift. The centre remained continuously operational during 2020–2021 and continued to provide emergency and inpatient HBOT under its usual clinical eligibility criteria; no pandemic-related closure or centre-level restriction on the acceptance of clinically indicated referrals was implemented.

#### Relationship to a Previous Publication

The patient cohort analysed in the present study corresponds to the ICU-HBOT cohort previously reported from the same centre by Miszewska et al. [1]. The previous publication examined safety-related factors during intra-ward transport and HBOT sessions and their associations with in-hospital mortality and length of hospitalisation. Accordingly, the overall cohort descriptors reproduced in Section 3.1 and Table 1 were reported previously [1], and are included here solely to define the cohort and contextualise the present secondary analysis. The new contributions of the present study are the calendar-year stratification of diagnostic composition, HBOT patient-session activity, session timing, catecholamine infusion, CVVHDF use, and in-hospital mortality, together with the exploratory calendar-time models presented in Section 3.2 and Section 3.3, Figure 2 and Table 2 and Table 3.

### 2.3. Study Population

The source population comprised all patients who received HBOT at the HC during the study period. The inclusion criteria were: (1) hospitalisation at the HC during the study period; (2) receipt of at least one HBOT session during that hospitalisation; and (3) a requirement for invasive mechanical ventilation via an endotracheal or tracheostomy tube. All included patients were managed in the HC’s inpatient intensive-care area. Eligibility was not restricted by diagnosis or HBOT indication, and no minimum number of HBOT sessions beyond one was required.

The exclusion criteria were: (1) receipt of HBOT exclusively in the outpatient setting; and (2) receipt of HBOT during hospitalisation without a requirement for invasive mechanical ventilation. No sampling procedure was applied. For annual analyses, each patient was assigned to the calendar year of admission to the HC.

### 2.4. Data Sources and Data Extraction

Data were extracted retrospectively from institutional medical records and departmental HBOT records. An HBOT patient-session was defined as one recorded HBOT treatment delivered to one patient. When two or more patients were treated during the same chamber run, each patient’s treatment was counted as a separate patient-session. For the analysis of treatment timing, patient-sessions starting between 19:00 and 07:00 were classified as nocturnal, and all remaining patient-sessions were classified as daytime.

Patient-level variables include age, sex, year of admission, length of hospitalisation at the HC, primary diagnosis or HBOT indication, total number of HBOT patient-sessions, number of daytime and nocturnal HBOT patient-sessions, catecholamine infusion, continuous veno-venous haemodiafiltration (CVVHDF), pleural drainage, and in-hospital mortality.

Diagnoses were assigned to four mutually exclusive categories: carbon monoxide poisoning; clostridial myonecrosis/gas gangrene; non-clostridial necrotising soft-tissue infections (other NSTIs, including Fournier’s gangrene); and other HBOT indications. Clostridial myonecrosis/gas gangrene was analysed separately from other NSTIs because of its distinct microbiological and clinical profile.

Aggregate service-level data were also extracted to describe the overall activity of the HC during the study period. These included the total number of HBOT-treated patients, the total number of HBOT patient-sessions, and the total number of hospitalised HBOT patients. These aggregate denominators were used only to contextualise the mechanically ventilated ICU-HBOT cohort within the overall HBOT service.

### 2.5. Outcomes

The primary outcome was the temporal change in diagnostic composition among mechanically ventilated ICU-HBOT patients across the eleven-year study period. Secondary outcomes included the annual patient volume, annual HBOT patient-session volume, the annual number and proportion of nocturnal HBOT patient-sessions, and calendar-time patterns in catecholamine infusion, CVVHDF use, and in-hospital mortality.

In-hospital mortality was defined as death during the index hospitalisation at the HC. Post-discharge mortality and long-term outcomes were not assessed.

### 2.6. Statistical Analysis

Continuous variables were summarised as medians with interquartile ranges, and categorical variables as counts and percentages. Annual numbers of patients, HBOT patient-sessions, nocturnal patient-sessions, diagnostic categories, organ-support variables, and in-hospital deaths were tabulated by calendar year. No formal trend test was applied to annual patient counts or annual HBOT patient-session counts; both were interpreted descriptively.

HBOT session timing was summarised using counts of nocturnal and daytime patient-sessions. Because individual patients could contribute more than one patient-session, these counts describe delivered HBOT activity rather than patient-level risk.

To supplement the descriptive annual data, calendar-time associations were estimated using exploratory univariable logistic regression models with the calendar year entered as a continuous predictor. Separate models were fitted for carbon monoxide poisoning, clostridial myonecrosis/gas gangrene, other NSTIs, other HBOT indications, catecholamine infusion, CVVHDF use, and in-hospital mortality. Model coefficients were exponentiated and reported as odds ratios per one-year increase with two-sided 95% Wald confidence intervals. The analysis used an exploratory estimation framework; results are therefore presented without *p*-values. The models were not interpreted as confirmatory analyses or as evidence of causal effects.

Data were complete for all variables included in the analyses. Analyses were performed in R version 4.3.3 with the following R packages: sjPlot, performance, report, gtsummary, MASS, and ggplot2. 

### 2.7. Ethical Approval

The study was conducted in accordance with the Declaration of Helsinki. The protocol was approved by the Independent Bioethics Committee for Scientific Research at the Medical University of Gdańsk, approval number: KB/52/2024, issued on 28 March 2024. Because of the retrospective design and use of existing medical records, the requirement for individual informed consent was waived by the Bioethics Committee.

## 3. Results

### 3.1. Overall Cohort Context and HBOT Activity

**Figure 1:**
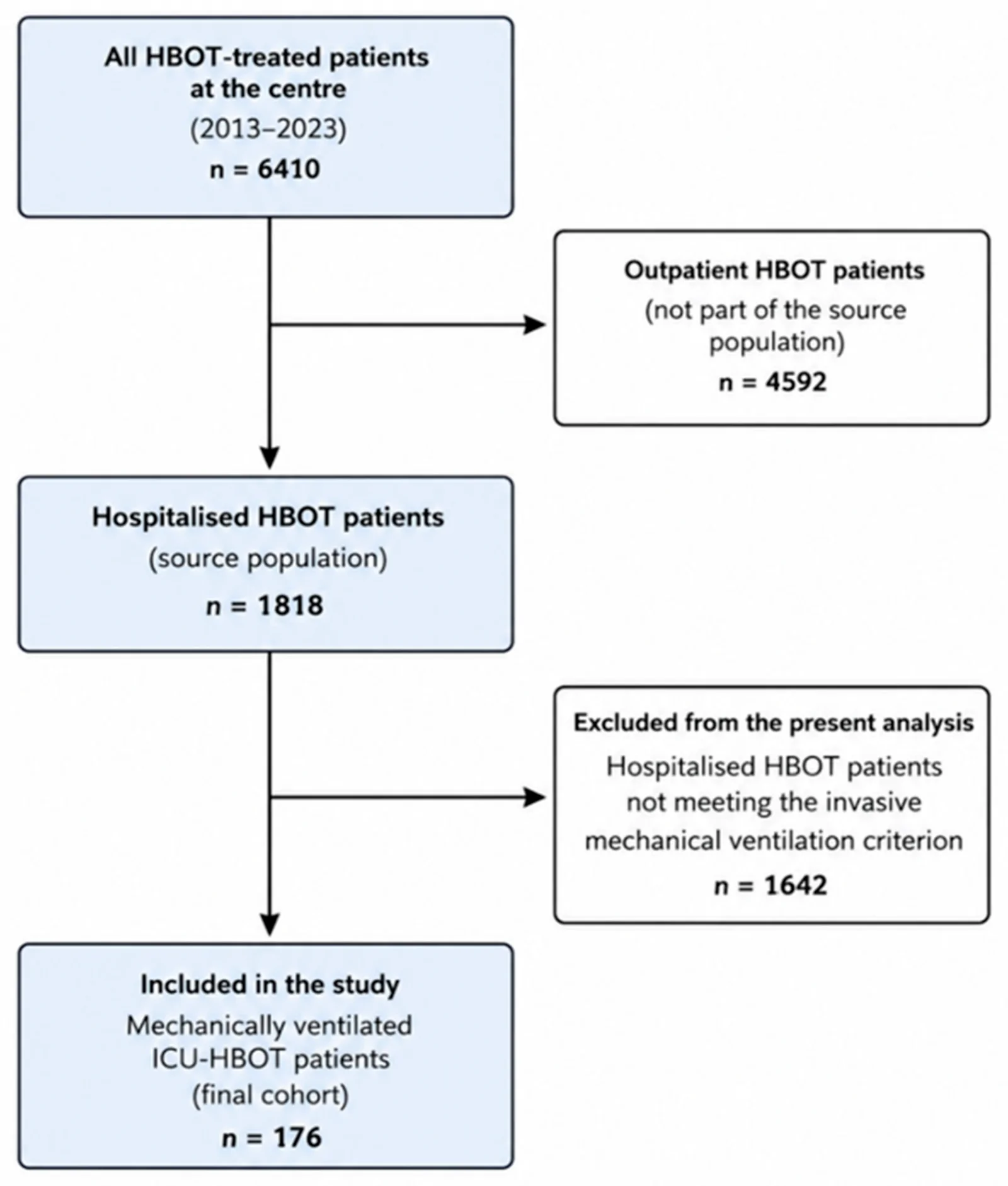
Flow diagram of study population selection. Abbreviations: HBOT, hyperbaric oxygen therapy; ICU, intensive care unit.

The cohort and its overall descriptive characteristics have been reported previously [1], and are summarised here solely to define the analytic cohort and contextualise the annual descriptive and exploratory calendar-time analyses that follow. During the study period, 6410 patients underwent HBOT at the HC, including 4592 outpatients and 1818 hospitalised patients. Among the hospitalised HBOT patients, 176 mechanically ventilated ICU-HBOT patients met the inclusion criteria. The selection of the study cohort is shown in Figure 1. Accordingly, ventilated ICU-HBOT patients accounted for 2.7% of all HBOT-treated patients and 9.7% of hospitalised HBOT patients [1].

The cohort comprised 127 men (72.2%) and 49 women (27.8%). Median age was 58 years [IQR: 41–67], median length of hospitalisation at the HC was 6 days [IQR: 3–8], and median number of HBOT sessions per patient was 8 [IQR: 3–11] [1]. Across the same period, the HC delivered 103,050 HBOT patient-sessions, of which 1381 (1.3%) were delivered to the mechanically ventilated ICU-HBOT cohort; 781 (56.6%) of the cohort’s patient-sessions were nocturnal. Catecholamine infusion was recorded in 130 patients (73.9%), CVVHDF in 37 patients (21.0%), pleural drainage in 11 patients (6.3%), and in-hospital death occurred in 28 patients (15.9%) [1].

**Figure 2:**
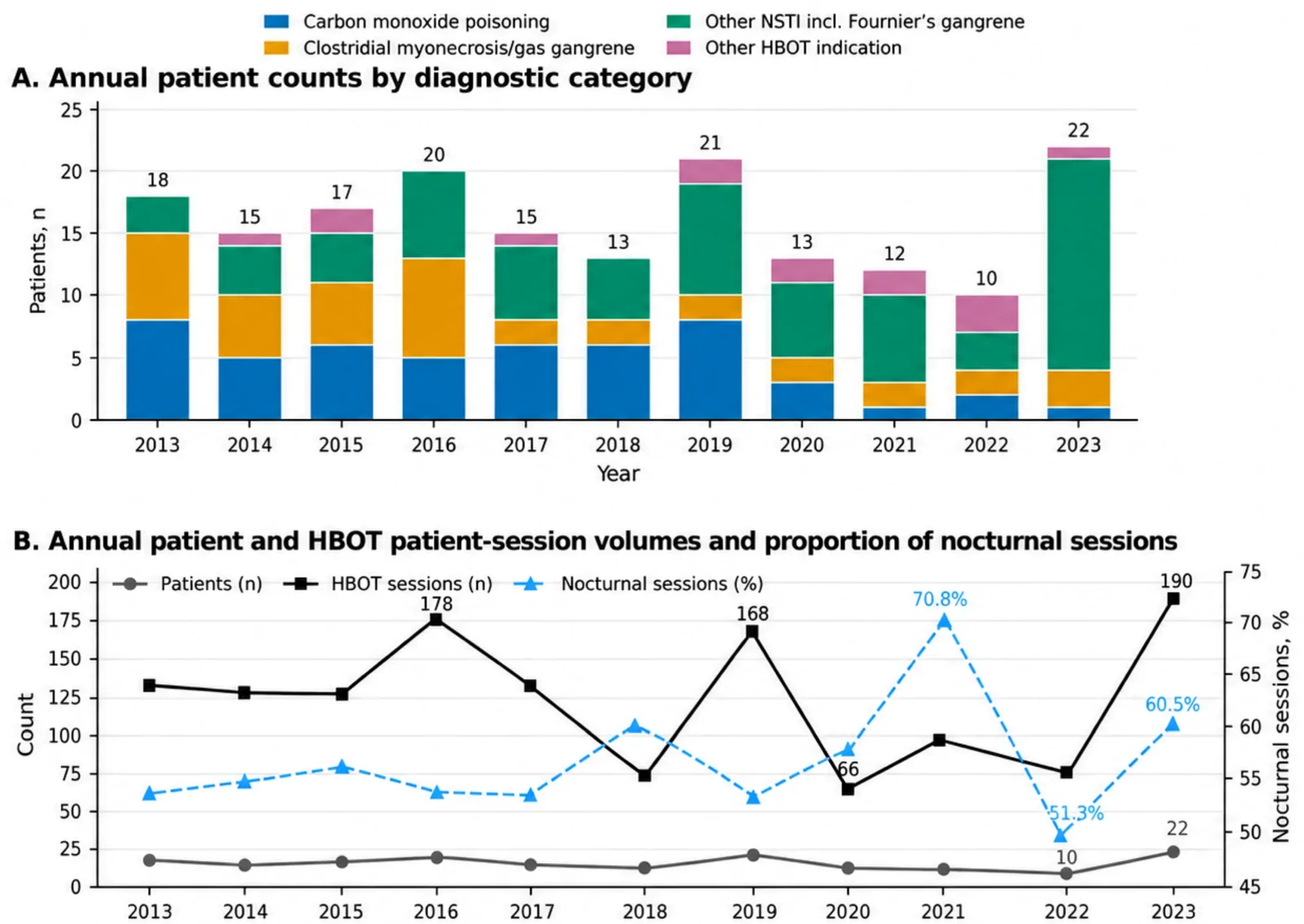
Annual diagnostic distribution and treatment activity among mechanically ventilated ICU patients treated with HBOT, 2013–2023. Panel (**A**) shows annual patient counts within each of the four mutually exclusive diagnostic categories. Panel (**B**) shows annual patient volume, HBOT patient-session volume, and the annual proportion of nocturnal HBOT patient-sessions. HBOT, hyperbaric oxygen therapy; ICU, intensive care unit; NSTIs, non-clostridial necrotising soft-tissue infections.

The most frequent diagnostic category was other NSTIs, including Fournier’s gangrene, recorded in 71 patients (40.3%), followed by carbon monoxide poisoning in 51 patients (29.0%), clostridial myonecrosis/gas gangrene in 40 patients (22.7%), and other HBOT indications in 14 patients (8.0%). The “other HBOT indications” category comprised gas embolism (*n* = 5), intracranial abscesses (*n* = 4), and one case each of decompression sickness, acute ischaemia, lung abscess, perispinal abscess, and mediastinal infection (Table 1 and Figure 2A).

**Table 1:** Demographic and clinical characteristics, diagnostic distribution, HBOT treatment activity, organ-support interventions, and in-hospital outcome of the mechanically ventilated ICU-HBOT cohort (*n* = 176). Previously published cohort descriptors are reproduced solely for context [1].

Variable	Value
**Demographic characteristics**	
Male sex, *n* (%)	127 (72.2)
Female sex, *n* (%)	49 (27.8)
Age, years, median [IQR]	58 [41–67]
**Diagnostic categories**	
Other NSTIs, including Fournier’s gangrene, *n* (%)	71 (40.3)
Carbon monoxide poisoning, *n* (%)	51 (29.0)
Clostridial myonecrosis/gas gangrene, *n* (%)	40 (22.7)
Other HBOT indications, *n* (%)	14 (8.0)
**Hospitalisation and HBOT treatment activity**	
Length of hospitalisation at HC, days, median [IQR]	6 [3–8]
HBOT sessions per patient, median [IQR]	8 [3–11]
Total HBOT patient-sessions, *n*	1381
Nocturnal HBOT patient-sessions, *n* (%)	781 (56.6)
**Organ-support interventions**	
Catecholamine infusion, *n* (%)	130 (73.9)
CVVHDF, *n* (%)	37 (21.0)
Pleural drainage, *n* (%)	11 (6.3)
**Clinical outcome**	
In-hospital mortality, *n* (%)	28 (15.9)

Data are presented as *n* (%) unless otherwise stated. Patient-level percentages were calculated using the 176 mechanically ventilated ICU-HBOT patients as the denominator. The percentage of nocturnal HBOT patient-sessions was calculated using the 1381 patient-sessions delivered to this cohort as the denominator. CVVHDF, continuous veno-venous haemodiafiltration; HBOT, hyperbaric oxygen therapy; HC, hyperbaric centre; ICU, intensive care unit; IQR, interquartile range; NSTIs, non-clostridial necrotising soft-tissue infections.

### 3.2. Annual Patient and HBOT Patient-Session Volumes, Nocturnal Patient-Sessions, and Distribution of Diagnostic Categories in the Mechanically Ventilated ICU-HBOT Cohort

Within the mechanically ventilated ICU-HBOT cohort, annual patient volume ranged from 10 patients in 2022 to 22 patients in 2023. Annual HBOT patient-session volume ranged from 66 sessions in 2020 to 190 sessions in 2023. The annual number of nocturnal sessions ranged from 39 (59.1% of all sessions in 2020) to 115 (60.5% in 2023); across calendar years, the proportion of nocturnal sessions ranged from 51.3% in 2022 to 70.8% in 2021. Percentages for nocturnal sessions were calculated using the number of HBOT sessions as the denominator. Diagnostic, organ-support, and mortality counts were patient-level counts (Table 2).

**Table 2:** Annual patient volume, HBOT patient-sessions, nocturnal patient-sessions, diagnostic categories, organ support, and in-hospital mortality.

Year	Patients, *n*	HBOT Patient-Sessions, *n*	Nocturnal Patient-Sessions, *n* (%)	CO, *n*	Clostridial Myonecrosis/ Gas Gangrene, *n*	Other NSTIs, Including Fournier’s Gangrene, *n*	Other, *n*	Catecholamine Infusion, *n*	CVVHDF, *n*	In-Hospital Mortality, *n*
2013	18	133	72 (54.1)	8	7	3	0	11	3	4
2014	15	128	70 (54.7)	5	5	4	1	10	7	2
2015	17	127	71 (55.9)	6	5	4	2	13	4	2
2016	20	178	96 (53.9)	5	8	7	0	14	3	4
2017	15	133	71 (53.4)	6	2	6	1	9	0	2
2018	13	82	49 (59.8)	6	2	5	0	10	1	3
2019	21	168	89 (53.0)	8	2	9	2	12	4	1
2020	13	66	39 (59.1)	3	2	6	2	10	2	2
2021	12	96	68 (70.8)	1	2	7	2	11	1	2
2022	10	80	41 (51.3)	2	2	3	3	9	2	3
2023	22	190	115 (60.5)	1	3	17	1	21	10	3
**Total**	**176**	**1381**	**781 (56.6)**	**51**	**40**	**71**	**14**	**130**	**37**	**28**

Values are counts unless otherwise stated. Nocturnal sessions are presented as *n* (%), and percentages were calculated using the annual number of HBOT sessions as the denominator. Diagnostic categories, catecholamine infusion, CVVHDF use, and in-hospital mortality are patient-level counts. No statistical testing was performed for this descriptive annual table. Abbreviations: CO, carbon monoxide poisoning; CVVHDF, continuous veno-venous haemodiafiltration; HBOT, hyperbaric oxygen therapy; NSTIs, non-clostridial necrotising soft-tissue infections; *n*, number of patients or patient-sessions, as applicable.

Descriptively, the annual data did not show a simple monotonic increase in patient or session volume. Instead, diagnostic composition changed across the study years: carbon monoxide poisoning and clostridial myonecrosis/gas gangrene were generally less represented in the later calendar years, whereas other NSTIs, including Fournier’s gangrene, were more prominent in several later years and accounted for 17 of 22 patients in 2023 (Figure 2A). Annual patient and HBOT patient-session volumes varied from year to year, and nocturnal sessions accounted for more than half of HBOT patient-sessions in every calendar year (Figure 2B).

### 3.3. Exploratory Associations of Calendar Year with Diagnostic Categories, Organ-Support Use, and In-Hospital Mortality

Exploratory univariable logistic regression models were fitted with calendar year entered as a continuous predictor (Table 3). The estimated OR per one-year increase was 0.85 (95% CI: 0.76–0.95) for carbon monoxide poisoning; 0.85 (95% CI: 0.76–0.96) for clostridial myonecrosis/gas gangrene; 1.23 (95% CI: 1.11–1.36) for other NSTIs; and 1.16 (95% CI: 0.97–1.39) for other HBOT indications.

**Table 3:** Exploratory univariable logistic regression models with calendar year as a continuous predictor.

Outcome	Events, *n*/N	OR per Year	95% CI
CO	51/176	0.85	0.76–0.95
Clostridial myonecrosis/gas gangrene	40/176	0.85	0.76–0.96
Other NSTIs, including Fournier’s gangrene	71/176	1.23	1.11–1.36
Other indication	14/176	1.16	0.97–1.39
Catecholamine infusion	130/176	1.17	1.04–1.31
CVVHDF	37/176	1.04	0.93–1.17
In-hospital mortality	28/176	0.99	0.87–1.12

Each row represents a separate exploratory univariable logistic regression model with calendar year entered as a continuous predictor. ORs are reported per one-year increase in calendar year; 95% CIs are two-sided Wald intervals. All estimates are unadjusted. The models estimate calendar-time associations within this single-centre cohort and were not used for confirmatory hypothesis testing or causal inference. Diagnostic categories were mutually exclusive. Abbreviations: CI, confidence interval; CO, carbon monoxide; CVVHDF, continuous veno-venous haemodiafiltration; HBOT, hyperbaric oxygen therapy; ICU, intensive care unit; *n*/N, number of events divided by the total number of mechanically ventilated ICU-HBOT patients; NSTIs, non-clostridial necrotising soft-tissue infections; OR, odds ratio.

For organ-support and outcome variables, the corresponding estimate was 1.17 (95% CI: 1.04–1.31) for catecholamine infusion; 1.04 (95% CI: 0.93–1.17) for CVVHDF use; and 0.99 (95% CI: 0.87–1.12) for in-hospital mortality. These estimates describe calendar-time associations within this cohort and do not imply causality.

## 4. Discussion

The main descriptive finding of this 11-year analysis is a change in the diagnostic composition of mechanically ventilated ICU patients treated with HBOT, rather than a simple increase in annual patient or HBOT patient-session volumes. Annual patient and HBOT patient-session volumes fluctuated, while carbon monoxide poisoning and clostridial myonecrosis/gas gangrene were generally less represented and non-clostridial NSTIs were more prominent in the later calendar years. The exploratory calendar-time estimates in Table 3 supplement these annual descriptions and should not be interpreted as confirmatory or causal effects. The operational burden remained substantial: all 176 patients required invasive mechanical ventilation; catecholamine infusion was recorded in 130 patients (73.9%); and 781 of 1381 HBOT patient-sessions (56.6%) were nocturnal. The clinically relevant observation is therefore a change in patient profile in the setting of sustained critical-care and service demands, rather than volume expansion alone.

This cohort should be interpreted against the background of contemporary HBOT practice, which is broad and mostly not focused on mechanically ventilated ICU patients. The International Multicenter Registry for Hyperbaric Oxygen Therapy included 22 centres and 2880 patients through June 2021, with delayed radiation injury, selected problem wounds, and CO poisoning among the most common UHMS-approved indications [12]. A subsequent registry analysis showed further diversification of HBOT practice, including emerging indications across several medical specialities [13]. Similarly, the Italian ITA-OTI prospective observational study described 327 consecutive HBOT patients from 10 centres, with most treatments being elective and the most frequent indications being sudden hearing loss, CO poisoning, and necrotising soft-tissue infection [14]. These studies are valuable because they show that HBOT is increasingly documented through multicentre real-world systems. However, they also highlight the specificity of the present cohort: all included patients were hospitalised, invasively mechanically ventilated, and required ICU-compatible HBOT. Thus, the present study addresses a narrow but operationally critical subgroup that is not adequately characterised by general HBOT registries.

The decreasing proportion of CO poisoning in this ventilated ICU-HBOT cohort is consistent with population-level reports from some high-income countries, but should be interpreted in the context of country-specific exposure patterns, prevention strategies, referral pathways, and HBOT availability. In Canada, mortality and hospital admission rates for unintentional non-fire-related CO poisoning declined steadily over time [15], and in England, hospital admissions for unintentional non-fire-related CO poisoning showed significant decreases after 2010 [16]. Local data from Gdynia provide centre-level support for an overall decline in CO poisoning treated in the hyperbaric service. In his PhD thesis, Góralczyk analysed 1672 patients admitted and treated for CO poisoning at the Department of Hyperbaric Medicine and Sea Rescue between 2009 and 2018, and reported annual case counts that decreased overall, despite year-to-year variability, from 203 cases in 2009, with a peak of 248 cases in 2010, to 122 cases in 2018 [17]. The present study concerns a more selected subgroup: CO-poisoned patients severe enough to require invasive mechanical ventilation and ICU-level HBOT. Therefore, the observed decline in CO within our cohort may reflect changes in exposure frequency, prevention, early detection, referral practice, or the severity distribution of CO poisoning. Residential CO alarms may facilitate earlier recognition of hazardous CO accumulation and termination of exposure; in an observational study of unintentional residential exposures, the absence of a CO alarm was associated with higher odds of CO poisoning and greater clinical severity [18]. However, alarm ownership and activation, exposure circumstances, and other preventive measures were not recorded in our study, and the contribution of residential alarms to the observed local trend cannot be determined. In Poland, a requirement covering specified rooms containing fuel-burning appliances was introduced only after the study period, in December 2024, with phased implementation for existing premises [19]. Conversely, national claims data from Korea showed an increasing prevalence of CO poisoning and increased HBOT provision between 2010 and 2019, underscoring that CO-related trends are country- and system-specific [20].

In contrast, non-clostridial NSTIs were the largest diagnostic category in several later calendar years. NSTIs are a rare but time-critical surgical emergency; recent reviews describe an increase in incidence during recent decades and emphasise the importance of clinical awareness and prompt recognition [21]. In a Danish nationwide registry study of 1527 patients with NSTIs from 2005 to 2018, incidence increased, mortality remained high, and ICU-level treatment was common: 99% of patients were admitted to an ICU, 86% were mechanically ventilated, 72% received vasopressor or inotropic support, and 18% underwent renal-replacement therapy [10]. These data illustrate the ICU-level organ-support burden commonly associated with NSTIs and provide external context for the increasing representation of NSTIs in the later calendar years. A Scandinavian prospective multicentre cohort similarly showed that most patients with NSTIs were managed in the ICU—frequently with mechanical ventilation and vasoactive support—and highlighted that haemodynamic instability and chamber capability can influence access to HBOT [22]. US administrative studies also document changing case profiles, high morbidity, and substantial system burden in NSTIs, although they are less directly comparable to our European ICU-HBOT setting [9,23].

From a service-planning perspective, the relevance of NSTIs lies not only in their frequency but also in the structure of care they generate. NSTI care commonly involves repeated surgical debridement, broad-spectrum antimicrobial therapy, ongoing organ-support management, and serial HBOT sessions. Published NSTI protocols may involve twice-daily HBOT for 72 h, three sessions within the first 24 h in selected anaerobic or gas gangrene presentations, and continuation when infection progression persists [22,24]. In the present cohort, the greater representation of NSTIs in the later calendar years occurred in the context of a high prevalence of catecholamine infusion and persistently high nocturnal session demand. Because nocturnal sessions were analysed at the session level, they describe the timing and volume of HBOT delivery rather than the patient-level risk. In this organisational model, repeated inpatient ICU-HBOT may require nocturnal delivery when daytime chamber capacity is occupied by routine outpatient HBOT. This interpretation is consistent with ICU-HBOT guidance requiring continuation of monitoring and critical-care treatment during HBOT, chamber-compatible equipment, and staff trained in both intensive care and hyperbaric medicine [2,5,7].

Findings for 2020–2021 should be interpreted in the context of the COVID-19 pandemic. Throughout this period, the centre remained continuously operational and did not suspend ICU-HBOT or impose pandemic-related restrictions on the acceptance of clinically eligible referrals. Patient and session volumes were nevertheless lower than in several pre-pandemic years, reflecting fewer incoming referrals rather than reduced local treatment availability. However, complete referral denominators and reasons for non-referral were not captured in the study dataset; therefore, changes in upstream referral and transfer pathways could not be quantified. The proportion of other NSTIs, including Fournier’s gangrene, was already high in 2020–2021, and the predominance of NSTIs persisted in 2022–2023. The COVID-19 pandemic may have influenced emergency presentation patterns, interhospital transfer, ICU capacity, operative pathways, and access to specialist services, but our study was not designed to test these mechanisms. External data support the plausibility of pandemic-related changes in NSTI pathways: Nguyen et al. found delayed hospital presentation among patients with necrotising fasciitis during the COVID-19 period but did not demonstrate major overall differences in ICU admission, length of stay, or mortality [25]. Accordingly, the present study can describe a local temporal pattern but cannot attribute it causally to the pandemic.

This study has several strengths. It includes eleven consecutive calendar years, a clearly defined ventilated ICU-HBOT population, and complete institutional capture of treatment sessions, including nocturnal activity. It combines patient-level clinical variables with service-level indicators, allowing interpretation of both epidemiological change and operational implications. The cohort is also relatively large for a single-centre series of mechanically ventilated ICU-HBOT patients, and complements prior safety-oriented publications by focusing on temporal trends. Several limitations must be acknowledged. This was a retrospective, single-centre study from a highly specialised academic hyperbaric centre with an integrated ICU-HBOT model; external validity may therefore be limited. The cohort included treated patients only and did not capture patients who were not referred, were considered too unstable for transfer, or had contraindications to HBOT. Referral and selection bias are therefore possible. Annual counts were small, and the logistic regression models were exploratory and univariable, assumed a constant linear association between calendar year and the log odds of each outcome, and did not adjust for potential confounding. Seven separate outcomes were modelled without multiplicity adjustment; therefore, the reported 95% confidence intervals are nominal and should be interpreted cautiously as centre-specific estimates rather than confirmatory or causal evidence of temporal change. Important severity and treatment variables were unavailable, including SOFA or APACHE scores, lactate, vasopressor dose, microbiology, extent of infection, timing and adequacy of surgical debridement, antimicrobial regimens, pre-transfer deterioration, and reasons for referral or non-referral. Mortality was limited to the index hospitalisation at the hyperbaric centre, and post-discharge mortality, neurological outcomes after CO poisoning, amputations, functional status, and quality of life were not assessed. Finally, although diagnoses were grouped into mutually exclusive categories, NSTI classification is clinically heterogeneous and may vary over time with coding practice, diagnostic awareness, and microbiological confirmation.

Future multicentre registries and prospective studies should include not only indications and outcomes, but also ICU-specific and service-level variables, such as mechanical ventilation mode, vasopressor dose, severity scores, timing of surgery and HBOT, nocturnal sessions, staffing model, transport events, and patient-centred outcomes. A logical next step would be to pool standardised ICU-HBOT and service-level data across critical care-capable hyperbaric centres and, where feasible, harmonise these data with the UHMS-supported Multicenter Registry for Hyperbaric Oxygen Therapy.

## 5. Conclusions

In this eleven-year single-centre cohort of mechanically ventilated ICU patients undergoing HBOT, annual diagnostic composition changed across the study period. Carbon monoxide poisoning and clostridial myonecrosis/gas gangrene were generally less represented, whereas non-clostridial necrotising soft-tissue infections, including Fournier’s gangrene, became more prominent in the later calendar years. Annual patient and HBOT patient-session volumes fluctuated without a monotonic increase.

Catecholamine infusion was recorded in 130 of 176 patients (73.9%), and 781 of 1381 HBOT patient-sessions (56.6%) were nocturnal. These findings support the need for ICU-capable hyperbaric infrastructure, chamber-compatible life-support equipment, multidisciplinary 24/7 staffing, and flexible scheduling models in centres providing emergency HBOT.

These centre-specific findings identify a service-relevant temporal pattern in a small but resource-intensive patient population and warrant validation in multicentre ICU-HBOT datasets.

## Data Availability

The data supporting the findings of this study are from the University Centre for Maritime and Tropical Medicine in Gdynia and are not publicly available. However, they can be obtained from the corresponding author upon reasonable request and with permission from the University Centre for Maritime and Tropical Medicine in Gdynia.

## Abbreviations

The following abbreviations are used in this manuscript:

APACHE	Acute Physiology and Chronic Health Evaluation
CI	Confidence interval
CO	Carbon monoxide
COVID-19	Coronavirus disease 2019
CVVHDF	Continuous veno-venous haemodiafiltration
HBOT	Hyperbaric oxygen therapy
HC	Hyperbaric centre
ICU	Intensive care unit
IQR	Interquartile range
NSTIs	Non-clostridial necrotising soft-tissue infections
OR	Odds ratio
SOFA	Sequential Organ Failure Assessment
UHMS	Undersea and Hyperbaric Medical Society
US	United States
